# Is Maxillomandibular Advancement Possible in Skeletal Class III Patients? A Scoping Review

**DOI:** 10.3390/jcm15030935

**Published:** 2026-01-23

**Authors:** Cheryl Ker Jia Lee, Jocelyn Kang Li Hor, Yi Lin Song, Raymond Chung Wen Wong, Crystal Shuk Jin Cheong, Chee Weng Yong

**Affiliations:** 1 Department of Orthodontics, National Dental Centre Singapore, Singapore 168938, Singapore; 2 Discipline of Oral and Maxillofacial Surgery, Faculty of Dentistry, National University of Singapore, Singapore 119085, Singapore; 3 Department of Otolaryngology—Head & Neck Surgery, National University Hospital Singapore, Singapore 119074, Singapore; 4 National University Centre for Oral Health Singapore, Singapore 119085, Singapore

**Keywords:** apnea, orthognathic surgery, sleep apnea syndromes, jaw, malocclusion angle class III

## Abstract

Unlike skeletal Class I and II patients, the application of maxillomandibular advancement (MMA) in skeletal Class III patients with obstructive sleep apnea (OSA) is not well documented. The aim of this scoping review was to explore the variations and outcomes of MMA techniques in this unique subgroup. A comprehensive search of PubMed, Embase, Cochrane and LILACS databases were conducted for articles published up to May 2025. Nine studies met the inclusion criteria. Three main variations of MMA were identified: (1) Bimaxillary advancement, which provides the most significant airway enlargement across all pharyngeal regions but carries the highest risk of facial aesthetic distortion; (2) Maxillary advancement with mandibular auto-rotation, a less invasive option suited for patients with isolated maxillary retrusion and symmetrical mandibular anatomy; (3) Maxillary advancement with mandibular setback, which addresses aesthetic concerns in patients with mandibular excess but may compromise oropharyngeal airway space. All variations were reported to be effective in treating OSA (Reduction of AHI by at least 50%) but with different considerations. Surgical planning for skeletal Class III patients with OSA should be individualized based on craniofacial morphology, anatomical site of airway obstruction, and aesthetic considerations. A decision flowchart was shared in this study.

## 1. Introduction

Maxillomandibular advancement (MMA) is a well-established and effective surgical intervention for the treatment of obstructive sleep apnea (OSA). It is frequently recommended as a primary surgical option for patients with dentofacial deformities, severe OSA, lateral pharyngeal wall collapse, or concentric velar collapse [1]. The procedure has demonstrated particularly high success rates in individuals with skeletal Class II malocclusion, where it is well understood that simultaneous advancement of the maxilla and mandible results in significant airway enlargement [2]. This leads not only to improved respiratory function but also favorable changes to the facial profile [3].

Modifications of MMA, collectively referred to as modified maxillomandibular advancement (MMMA), have been proposed to address potential aesthetic concerns associated with the standard procedure. Yong et al. described variations such as combining MMA with anterior segmental osteotomy to mitigate post-operative facial fullness [4]. These approaches are especially relevant for patients with bimaxillary protrusion, where additional skeletal advancement may lead to an unesthetic facial profile. This consideration is particularly important in certain populations such as East Asians, where bimaxillary protrusion is more prevalent. This highlights the need for individualized surgical planning [4,5,6,7].

In contrast, the literature on the surgical management of OSA in patients with skeletal Class III patterns remains limited, and there remains uncertainty regarding the most effective approaches. These patients typically present with a hypoplastic maxilla and hyperplastic mandible. There is a with a higher prevalence of Class III malocclusion among East Asian populations compared with Western populations [8]. Kaewja et al. reported that 11.22% of skeletal Class III orthognathic surgery patients were diagnosed with obstructive sleep apnea (OSA) preoperatively [9]. Similarly, Kim et al. analyzed skeletal profiles of 1226 patients diagnosed with OSA and found that 10.5% exhibited a skeletal Class III relationship [10]. These patients were frequently noted to have a narrower nasopharyngeal airway space compared to individuals with other dentofacial patterns [10].

Orthognathic surgery in this group of patients often involves mandibular setback procedures with the goal of addressing occlusion and/or facial profile concerns. This is seemingly counterintuitive to the goal of OSA surgery, which is to enlarge the airway. While existing studies suggest that bimaxillary surgery (maxillary advancement combined with mandibular setback) does not elevate the risk of developing OSA postoperatively, there is a paucity of data focusing specifically on patients diagnosed with OSA prior to surgery [11]. This gap in knowledge underscores the need for further research into tailored surgical approaches for managing OSA in skeletal Class III individuals.

In the revised Stanford protocol proposed by Liu et al. in 2019, it was recommended that patients with skeletal Class III deformities should not routinely undergo standard maxillomandibular advancement (MMA) [1]. Instead, they advocated for counterclockwise rotation and advancement of the maxilla, paired with rotational repositioning of the mandible, thereby eliminating the need for mandibular setback. This highlights a key clinical dilemma: although isolated maxillary advancement may improve airway patency, it may limit aesthetic improvement by failing to address underlying mandibular prognathism. Conversely, mandibular setback may preserve facial harmony but risks compromising airway volume and exacerbating OSA symptoms.

Given these unique anatomical and aesthetic challenges in Skeletal Class III patients, careful surgical planning is essential. An integration of current knowledge is hence warranted to evaluate the effectiveness, safety, and most appropriate surgical approaches of MMA in OSA among this unique patient population. This study aims to systematically identify and synthesize existing primary research on the use of MMA, including its various modifications, in treating skeletal Class III patients diagnosed with OSA.

## 2. Materials and Methods

A scoping review was conducted to map and synthesize the evidence on maxillomandibular advancement (MMA) in skeletal Class III patients with obstructive sleep apnoea, an area characterised by heterogeneous surgical approaches, study designs, and outcome measures. This approach was selected to identify key concepts, variations in surgical techniques, and assess their effectiveness. Effectiveness or surgical success was defined as reduction in apnea–hypopnea index (AHI) by at least 50% [12].

The protocol for this review was created according to the Joanna Briggs Institute scoping review protocol guidelines, and was performed using the five steps proposed by Arksey and O’Malley [13,14]. PRISMA-ScR guidelines were used to guide the reporting of this review [15]. The protocol of this review is available in the INPLASY Register (INPLASY20256006). Ethical approval was not required for the study.

### 2.1. Research Question

The primary research question was: “How is maxillomandibular advancement (MMA) performed in skeletal Class III patients diagnosed with obstructive sleep apnea (OSA)?”

Based on this, the objectives of the study were to:

1. Identify the surgical techniques and approaches used in MMA for this patient group.
2. Examine the clinical considerations influencing treatment selection.

### 2.2. Search Strategy

A systematic search was conducted in PubMed, Embase, Cochrane Library and LILACS using a comprehensive set of keywords, including: maxillomandibular advancement (MMA), maxillary advancement, modified maxillomandibular advancement (MMMA), bimaxillary advancement, telegnathic surgery, bimaxillary rotation, counterclockwise rotation (CCWR), sub-apical osteotomy, prognathism, progenia, Class III, Class 3, mandibular hyperplasia, obstructive sleep apnea (OSA), sleep-disordered breathing, apnea–hypopnea index (AHI), respiratory disturbance index (RDI), oxygen desaturation index (ODI), and lowest oxygen saturation (LSAT) (Supplementary Material File S1). References of relevant review articles were also screened to identify additional eligible studies. The final search was completed on 1 May 2025. There was no language restriction applied to the selection of the studies.

### 2.3. Study Selection

Two independent reviewers (CWY and CLKJ) screened the titles and abstracts based on relevance to the research question. Full-text articles were then assessed for eligibility.

**Inclusion criteria** were:

1. Primary research articles
2. Patients with skeletal Class III deformities (ANB angle < 2°)
3. Use of MMA or skeletal advancement surgery specifically for the treatment of OSA
4. Patients diagnosed with OSA

**Exclusion criteria** were:

1. Review articles
2. Absence of skeletal Class III patients with OSA
3. Inability to isolate data specific to Class III patients
4. No skeletal advancement or MMA performed
5. Lack of full-text availability
6. Presence of craniofacial syndromes

A third reviewer was involved if there were differing opinions between the two main reviewers (HKLJ).

### 2.4. Data Extraction

Data were extracted independently by two reviewers (CWY and LKJC) using a standardized template. A third reviewer (JKLH) resolved any discrepancies between the two primary extractors. Extracted data included author, year of publication, journal, population studied, sample size, sex, mean age, mean body mass index (BMI), MMMA surgical technique, surgical movements, and outcome measures. The outcome measures included the apnea–hypopnea index (AHI), Epworth sleepiness scale (ESS) score, radiographic changes to airway dimensions, aesthetics, complications, or any other quality of life measures.

### 2.5. Analysis and Synthesis of Results

Cohen’s kappa statistic was used to assess inter-rater agreement. A descriptive synthesis of the included studies was performed due to heterogeneity in study design, surgical techniques, and outcome measures.

## 3. Results

**Figure 1:**
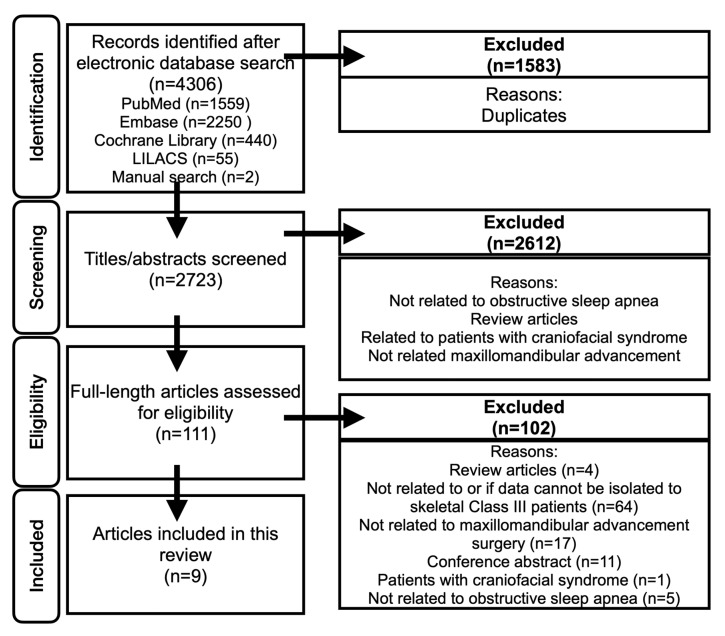
PRISMA-Scr Flowchart.

The search of four databases yielded 4307 articles. 1584 duplicated articles were eliminated. The titles and abstracts of 2723 articles were screened (inter-rating κ = 0.91). A total of 111 full-length articles were reviewed. A final 9 articles were included in this scoping review (Figure 1), and the extracted data are summarized in Table 1 and Table 2. The inter-rating agreement was high (κ = 0.89).

### 3.1. Patient Demographics

The nine included studies reported 27 skeletal Class III patients, predominantly male (92.6%), with a mean age of 38.2 ± 13.4 years and a mean BMI of 28.8 ± 7.5 kg/m^2^. Baseline OSA severity was generally moderate to severe (mean AHI/RDI 42.7 ± 31.7 events/h). Other baseline measures, including ESS (10.9 ± 3.9; *n* = 20), ODI (17.3 ± 20.3 events/h; *n* = 16), and posterior airway space (6.5 ± 2.1 mm; *n* = 9), were reported in small subsets only and should therefore be interpreted with caution. With the exception of Alsaty et al. (2-month follow-up), all other studies reported follow-up periods of 6–24 months [19].

### 3.2. Methodological Analysis

All included studies met reporting standards based on the CARE guidelines for case reports and the Joanna Briggs Institute checklist. However, the overall quality of evidence remains limited. Sample sizes were small, reducing statistical power. Patient selection was non-randomised, introducing a risk of bias that was further compounded by the retrospective nature of most studies. In addition, with the exception of Abdelwahab et al., comparison or control groups were lacking, limiting the ability to attribute observed outcomes directly to the intervention.

### 3.3. Description of the Variations of MMA

Across the included studies, three variations of MMA or skeletal advancement surgery were reported in the management of skeletal Class III patients with OSA (Table 2).

#### 3.3.1. Variation 1: Maxillary and Mandibular Advancement

**Figure 2:**
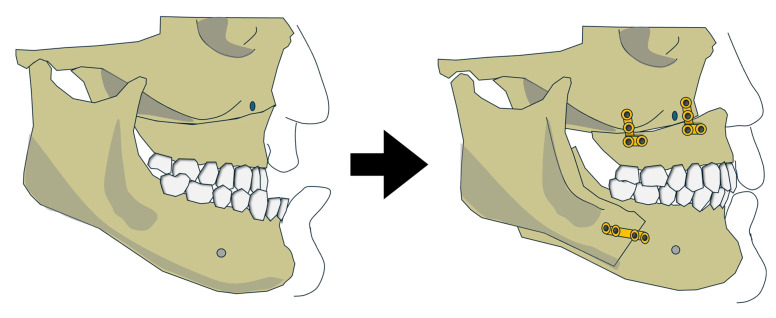
Variation 1 before and after the surgery.

Five studies described the use of conventional MMA in skeletal Class III OSA patients (Figure 2) [16,17,18,19,20]. However, data isolating outcomes specifically for Class III patients were available for only 10 individuals, all from predominantly White populations.

The baseline cephalometric values were as follows: Sella-Nasion-A point (SNA) = 80.04 ± 5.26°, and Sella-Nasion-B point (SNB) = 81.01 ± 2.14°. Among the included studies, only Alsaty et al. reported the extent of maxillary advancement (9 mm) [19]. Across the pooled data, the mean changes in SNA and SNB were 9.09 ± 4.04° and 5.11 ± 2.27°, respectively. The average reduction in apnea–hypopnea index (AHI) or respiratory disturbance index (RDI) was 85.24 ± 10.84%.

Within-study comparisons indicated mixed outcomes. Brevi et al. reported a slightly lower AHI reduction in Class III patients (78.52 ± 7.61%, *n* = 2) compared to Class I and II patients (81.45 ± 9.81%, *n* = 30) [18]. In contrast, Ronchi et al. found similar AHI reductions in Class III (86.50 ± 9.63%, *n* = 3) and Class I/II patients (86.03 ± 8.33%, *n* = 19) [17]. Li et al. observed greater efficacy in Class III patients (91.58 ± 5.53%, *n* = 3) versus Class I/II patients (87.12 ± 14.37%, *n* = 20) [20].

ESS scores were reported in six patients, with a mean reduction of 10 ± 5. Notably, one patient showed no change (ESS 8 to 8), while the remaining patients exhibited significant improvement. PAS enlargement was noted in 6 patients, with an average increase of 79.68 ± 52.16% [16,17,18,20]. Data on ODI were reported only by Brevi et al. (*n* = 2), which decreased from 32 to 4 and 84 to 9, respectively [18].

Complications were reported in three patients undergoing bimaxillary advancement (variation 1). One 26-year-old patient reported worsened facial aesthetics postoperatively (Grade “C” on an aesthetic satisfaction questionnaire), although no details regarding subsequent management were provided. Another patient, a 47-year-old female, required reoperation due to skeletal instability resulting from inadequate fixation. The last patient was a 48-year-old male who had his mandibular fixation implants removed due to localized irritation [17,20]. The timing of these complications was not specified. Although all three patients exhibited relatively larger changes in SNA (and SNB in one case), this could not be confirmed as a risk factor. No late complications were reported.

#### 3.3.2. Variation 2: Maxillary Advancement with Mandible Auto-Rotation

Two case reports described the use of isolated maxillary advancement with mandible auto-rotation in the management of Class III patients with OSA [21,22]. The available data is limited to individual case descriptions and therefore represents a very low level of evidence.

**Figure 3:**
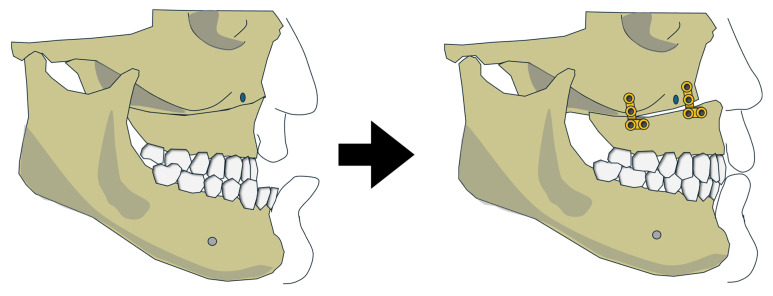
Variation 2 before and after the surgery.

Ishida et al. (2019) described a Japanese patient treated with isolated maxillary advancement (4.5 mm at the anterior nasal spine), resulting in mandibular auto-rotation due to superior repositioning of the maxilla (Figure 3) [22]. Postoperative SNA increased from 74.6° to 77.8°, while SNB remained unchanged at 77.1°. AHI was reduced from 15.3 to 2.8 events/h, reflecting an 81.7% improvement. The authors suggested this approach may be preferable for patients with Class III deformities due to maxillary retrusion but was not suitable for those with severe mandibular asymmetry or canting. Hoshijima et al. (2015) describe the same approach for another Japanese patient [21]. Similarly, the maxillary advancement was modest, with only 3.5 mm at the anterior nasal spine. Both the postoperative SNA and SNB increased from 80.5° to 84.5° and 80.5° to 82.0°, respectively. AHI was reduced from 18.8 to 7.6 events/h (59.6%). No complications were reported for both patients. While both reports showed improvement in OSA-related outcomes, the findings are based on isolated cases and should be interpreted with caution.

#### 3.3.3. Variation 3: Maxillary Advancement and Mandibular Setback

Lu et al. reported the treatment of a class III patient with moderate OSA (AHI 22.8 events/h) with maxillary advancement and mandibular setback [24]. The maxillary anterior and mandibular posterior movements were equal at 4 mm. In addition, counterclockwise rotation of the maxillomandibular complex and genioplasty advancement may have further facilitated the improvement in AHI (55.7% reduction).

**Figure 4:**
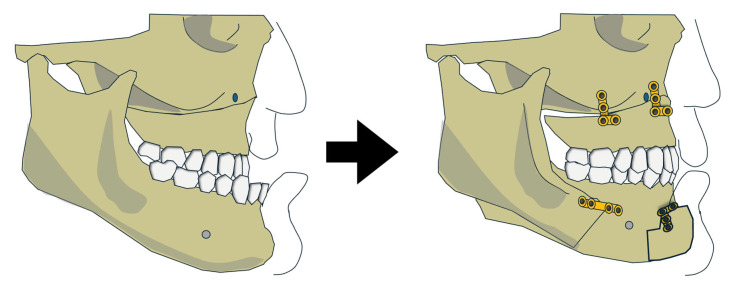
Variation 3 before and after the surgery.

A retrospective study by Abdelwahab et al. (2023) reported outcomes from 14 skeletal Class III patients (10 White, 4 non-White) treated with maxillary advancement combined with mandibular setback (Figure 4) [23]. Preoperative SNA and SNB were not defined, and only mean postoperative values were reported: SNA = 80.69°, SNB = 82.72°. The extent of surgical movements was similarly not detailed, but a surgical simulation figure suggested an 8.3 mm overlap between proximal and distal mandibular segments at the inferior border. The utilisation of advancement genioplasty and rotation of the maxillomandibular complex were mentioned but not well described.

Mean AHI decreased from 37.17 to 11.81 events/h, reflecting a 68.23% reduction in OSA severity. Surgical success was achieved in 78.6% of skeletal Class III patients, with a cure rate of 35.7%. The skeletal Class II cohort had a higher success rate (92.8%) but a lower cure rate (21.4%). Although the authors described outcomes as comparable between groups, surgical success was lower in skeletal Class III patients. Hence, these findings should be interpreted with caution.

The authors looked at outcomes in the skeletal class III OSA patients with pre-operative SNB < 80° versus SNB > 80° and found that there was no statistical differences in the preoperative AHI and change in AHI. There was similarly no statistical difference in preoperative AHI and change in AHI between patients with preoperative SNA < 82° versus SNA > 82°.

Additional reported outcomes included reductions in ESS (from 10.23 ± 4.38 to 4.22 ± 3.07) and ODI (from 11.43 ± 11.40 to 5.44 ± 7.96). No surgical complications were reported.

### 3.4. Comparisons Between Variations

**Figure 5:**
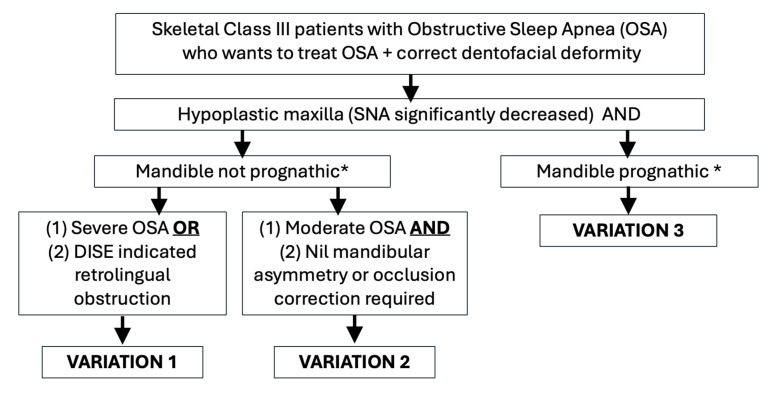
Flowchart on decision process. * Prognathism is determined by SNB values on cephalometric analysis or if prognathism has affected aesthetics.

All patients achieved treatment success, with AHI reductions ranging from 55.7% (variation 3) to 98.57% (variation 1). Variation 1 demonstrated the greatest absolute reduction in AHI, with a mean decrease of 49.88 events/h (85.24%). Variations 2 and 3 showed similar percentage reductions in AHI (approximately 70%). However, the absolute postoperative AHI remained higher in variation 3 (approximately 11 events/h) compared with variation 2 (approximately 5 events/h).A flowchart recommending the selection of a treatment variation is illustrated in Figure 5.

## 4. Discussion

Maxillomandibular advancement (MMA) is a well-established surgical treatment for obstructive sleep apnea (OSA) in patients with skeletal Class I and II relationships. However, its efficacy in skeletal Class III OSA patients remains less clear. This scoping review identified and analyzed nine studies involving 27 skeletal Class III OSA patients, revealing three distinct surgical variations of MMA utilized in this subgroup. The findings underscore the necessity of individualized surgical planning to optimize both functional and aesthetic outcomes. Based on the results from the studies, a decision-making flowchart was generated.

### 4.1. Variation 1: Bimaxillary Advancement

While the forward projection of the chin in skeletal Class III patients could potentially compromise facial aesthetics, no such concerns were reported. Several factors may explain this: (1) the average pre-treatment SNB angle was not significantly high (81.3 ± 2.36°), indicating maxillary retrusion rather than mandibular prognathism as the dominant deformity; (2) although bimaxillary advancement was done, the SNA change (8.70 ± 3.74°) exceeded that of SNB (5.01 ± 2.64°), supporting the notion that maxillary advancement played a larger role in the correction; and (3) the patient population was predominantly White, a group known to tolerate or aesthetically accommodate bimaxillary advancement due to baseline retrusive profiles [25]. All the patients included in the studies had severe pre-treatment OSA. While oropharyngeal obstruction was also reported for 1 patient (endoscopy findings not reported for other patients), the need to further expand the mandibular framework should also be considered for patients oropharyngeal obstruction.

Therefore, based on the limited available evidence and small case numbers, variation 1 may be considered for selected skeletal Class III patients with OSA, particularly those without marked mandibular prognathism, with severe OSA, or with predominant oropharyngeal obstruction.

### 4.2. Variation 2: Isolated Maxillary Advancement with Mandibular Auto-Rotation

The second variation features isolated maxillary advancement and impaction. This induces a counter-clockwise rotation of the mandible, leading to an automatic enlargement of the upper airway. This technique is particularly beneficial for patients with maxillary retrusion and no significant cants or mandibular asymmetry.

Before selecting this surgical variation, it is essential to accurately identify the primary site of airway obstruction. This approach may be particularly beneficial for patients with velopharyngeal or nasopharyngeal narrowing. Studies have shown that maxillary advancement alone can significantly increase upper airway volume, including both the nasopharyngeal and oropharyngeal regions. Rosário et al. reported increased upper airway volume following isolated maxillary advancement in patients with skeletal Class III deformities, and a systematic review by Steegman et al. similarly demonstrated volumetric airway expansion extending into the oropharynx [26,27]. However, the degree of airway enlargement is generally less than that achieved with bimaxillary advancement, which produced more substantial changes particularly in the oropharyngeal space.

These findings were derived from non-OSA populations and should therefore be extrapolated to OSA patients with caution. Importantly, anatomical changes alone do not fully reflect OSA pathophysiology, which is also influenced by airway collapsibility and neuromuscular control [28].

Based on the limited findings of the study, variation 2 may be considered only in carefully selected patients with moderate OSA who do not require mandibular surgery for correction of facial asymmetry or occlusal discrepancies. However, the role of isolated maxillary advancement in the management of OSA still requires further validation, especially given the small sample sizes and limited population-specific data.

### 4.3. Variation 3: Maxillary Advancement with Mandibular Setback

The third variation entailed a combination of maxillary advancement and mandibular setback, a strategy traditionally used in non-OSA Class III patients for facial balance. Despite concerns that mandibular setback could reduce the oropharyngeal airway and exacerbate OSA, surgical success rates remained high. This suggests that maxillary advancement may offset the airway narrowing effect of the mandibular setback, possibly by advancing the velopharyngeal musculature and soft tissue. Some studies propose that reductions in airway volume following mandibular setback are transient, with subsequent remodelling and rebound over time [11,29,30]. Additionally, skeletal Class III patients may possess an enlarged posterior airway pre-operatively, allowing a margin of reduction without significant physiological consequence [11,29,30].

However, none of the reviewed studies included precise data on pre-treatment airway volumes or obstruction sites. Therefore, predictability of MMA for Class III patients remains uncertain. The heterogeneity of skeletal Class III deformities—whether due to maxillary retrusion or mandibular prognathism—did not appear to significantly affect surgical outcomes, though further investigation is warranted to clarify this relationship. As with variation 2, it remains unclear whether airway changes in non-OSA patients respond similarly to those in OSA patients. Therefore, identifying the pre-treatment site of obstruction and assessing airway volume are important for determining the appropriateness of this surgical approach.

A recent systematic review by Grinberg et al. evaluated risk of airway compromise following orthognathic surgery in Class III patients [31]. The findings of the study challenged prior perspectives that bimaxillary surgeries (maxillary advancement and mandibular setback) led to better oropharyngeal airway volume preservation than mandibular setback alone. In another systematic review by Steegman et al., the authors investigated the three-dimensional airway volume changes after various orthognathic surgeries [27]. It was reported that bimaxillary advancement and isolated maxillary advancement were more consistently associated with airway enlargement. In comparison, maxillary advancement with mandibular setback may either expand or shrink the airway. These findings indicate that although variation 3 may be appropriate for patients with true mandibular prognathism, the oropharyngeal airway remains at risk. Careful case selection is therefore essential, and adjunctive procedures such as counterclockwise rotation or genioglossus advancement should be considered when appropriate.

Previous reviews have reported lower apnea–hypopnea index (AHI) reductions in Class III patients undergoing MMA compared to Class I and II patients, hypothesizing that less mandibular advancement may underlie this trend [32]. However, our review did not corroborate this, as MMA outcomes ranged from superior to comparable or inferior. This suggests that factors beyond the extent of mandibular advancement, such as individual craniofacial morphology and surgical technique, play critical roles. Thus, results of this review highlights that MMA in Class III patients cannot be standardized but should instead be tailored to the individual’s anatomical and functional needs.

Surgeons often target 10 mm of maxillary and mandibular advancement based on earlier reports [3]. While this benchmark may serve as a guideline, the degree, direction, and type of skeletal movement must consider disease severity, anatomical site(s) of obstruction, and the patient’s baseline craniofacial structure. The decision-making process in Class III patients is particularly complex, as it may involve trade-offs between airway patency and aesthetic balance, particularly in cases where mandibular setback is contemplated.

Beyond jaw repositioning, mandibular plane angle is a relevant cephalometric variable linked to OSA severity and treatment response. High-angle mandibles are associated with narrower oropharyngeal airways and reduced response to therapy [33,34]. Ma et al. reported that patients with a high mandibular angle have a smaller baseline dimension of the oropharyngeal airway and required a greater protrusion to achieve an effective treatment [34]. While this factor was not consistently reported in the included studies, it should be integrated into pre-operative assessment and planning.

Advancements in digital technology has allowed the creation of a digital twin for virtual surgical planning (VSP), computed aided design and 3D printing [35,36]. The improvements such as greater accuracy, patient safety, improved simulations have been demonstrated in orthognathic surgery patients [35,36]. These same improvements were shown to be translated to MMA. The use of VSP has been shown to be feasible and safe while allowing predictable aesthetic and functional changes in MMA patients [37]. Greater precision could be achieved with the use of surgical guides and customized fixation devices [38].

The future of MMA planning may also benefit from predictive simulations of airway response. Computational fluid dynamics (CFD) has demonstrated promise in modelling airway behaviour post-surgery and may help optimize surgical planning by quantifying expected changes in airflow dynamics [39]. A target of 70% airway enlargement has been suggested, but tools to accurately simulate these changes preoperatively are still in development and not widely accessible [40].

This review has several limitations. First, the small sample size and heterogeneous methodologies across the included studies precluded formal meta-analysis. The current evidence is largely derived from case reports and small case series, where limited methodological control and absence of standardised protocols increase susceptibility to bias and reduce generalisability. In addition, inconsistent assessment and reporting of key surgical parameters, particularly mandibular plane angle and site of airway obstruction, limit comparability across studies and constrain evidence-based surgical decision-making. Next, most studies lacked detailed assessment and description of the specific sites of upper airway obstruction, which limits personalized surgical recommendations. Only 1 study described the patient to have both velopharyngeal and oropharyngeal obstruction [16]. Lastly, while SNA and SNB changes were consistently reported, linear advancement in millimetres and rotational movements were less frequently documented—metrics that many clinicians find more practical for surgical planning. Finally, the studies reviewed primarily included White populations, limiting generalizability to other ethnic groups who may have different aesthetic thresholds and craniofacial morphology.

This scoping review consolidates available evidence on MMA variations in skeletal Class III patients with OSA. Although no universal protocol can be recommended, the findings support a nuanced, individualized approach to surgical planning. Importantly, this review also highlights the relative paucity of research in this specific population—one that may be underrepresented in the current OSA surgical literature. Further prospective studies with standardized reporting on anatomical, functional, and aesthetic outcomes are urgently needed to guide evidence-based surgical management in this subgroup.

In conclusion, this scoping review highlights that while MMA is effective in achieving surgical success in OSA (AHI reduction by at least 50%), its use in skeletal Class III patients requires individualized planning. Three surgical variations (bimaxillary advancement, isolated maxillary advancement with mandibular autorotation, and maxillary advancement with mandibular setback) were identified, each offering potential benefits depending on facial morphology, site of airway obstruction, and aesthetic considerations. The findings emphasize that treatment cannot rely on standardized protocols but must instead be tailored to patient-specific anatomy and functional needs. However, all proposed approaches are derived from limited evidence and should be applied cautiously, with strategic case selection rather than as standardised treatment protocols. In particular, pre-treatment assessment of oropharyngeal obstruction is critical, as certain approaches may risk worsening airway compromise. Future prospective studies with larger cohorts and standardised outcome measures are required to validate these strategies and to guide evidence-based management in skeletal Class III OSA patients.

## Figures and Tables

**Table 1:** Demographic details and surgical movements.

Author & Year	Study Type	Sample Size	Mean Age (Years)	Gender	BMI (kg/m^2^)	SNA, SNB and Surgical Movements
Variation 1: Maxillary and mandibular advancement						
Rossi et al. 2019 (Patient 6) [16]	Retrospective Cohort	1	47	M	30	SNA: 75.9° → 81.6°, (Dx + 5.7°) SNB 79.3° → 84.9°, (Dx + 5.6°) Maxilla advancement not noted in mm, Mandible advancement 13.2 mm
Ronchi et al. 2010 (Patients No. 5 from group 1, No. 5 and 6 from group 2) [17]	Retrospective Cohort	3	26	M	24	SNA: 73° → 89°, (Dx + 16°) SNB 79° →85°, (Dx + 6°)
			62	M	27.1	SNA: 80° → 85°, (Dx + 5°) SNB 80° → 84°, (Dx + 4°)
			54	M	31	SNA: 82° → 91°, (Dx + 9°) SNB 82 → 88, (Dx + 6°)
Brevi et al. 2011 (Patients No. 1, 6) [18]	Retrospective Cohort	2	67	M	25.7	SNA 79° → 86°, (Dx + 7°) SNB 84° → 88° (Dx + 4°) Movements not recorded in mm
			45	M	30	SNA 75° → 85°, (Dx + 10°) SNB 78° → 82°, (Dx + 4°)
Alsaty et al. 2021 [19]	Case Report	1	55	M	25.6	SNA 78.5° → 83.7°,(Dx + 5.2°) SNB 78.8° →81.3°, (Dx + 2.5°) Maxilla advancement 9 mm, Mandible advancement 5 mm
Li et al. 2000 (Patients No. 7, 13, 21) [20]	Retrospective Cohort	3	47	F	56.0	SNA 83° → 96°, (Dx + 13°) SNB 83° → 88° (Dx + 5°)
			17	M	41.5	SNA 83° → 88°, (Dx + 5°) SNB 84° → 87°, (Dx + 3°)
			48	M	42.1	SNA 81° → 96° (Dx + 15°) SNB 82°→ 93° (Dx + 11°)
Variation 2: Maxillary advancement with mandible auto-rotation						
Hoshijima et al. 2015 [21]	Case Report	1	44	M	NA	SNA 80.5° → 84.5°, (Dx + 4.0°) SNB 80.5° → 82.0°, (Dx + 1.5°) 3.0 mm forward at ANS
Ishida et al. 2019 [22]	Case Report	1	23	M	18.6	SNA 74.6° → 77.8° (Dx + 3.2°), SNB 77.1° → 77.1° (Dx 0) 4.5 mm forward at ANS
Variation 3: Maxillary advancement and mandibular setback						
Abdelwahab et al. 2023 [23]	Retrospective Cohort	14	33.9 ± 10.2	13 M, 1 F	26.9 ± 3.7	Post-op SNA 80.69° Post-op SNB 82.72° Surgical movements not defined
Lu et al. 2024 [24]	Case Report	1	24	1 M	21.7	SNA 86.1° → 88.6°(Dx + 2.5°), SNB 88.6° → 88.1°(Dx − 0.5°), Maxilla advancement 4 mm & counterclockwise rotation of 5°, mandible setback 4 mm & counterclockwise rotation of 6.5°, genioplasty advancement 7 mm

Legend: BMI = Body mass Index, M = Male, F = Female, Dx = Difference of, SNA = Sella-Nasion-A Point Angle, SNB = Sella-Nasion-B Point Angle.

**Table 2:** Outcomes.

Author & Year	AHI/RDI (Events/h)			ESS			ODI (Events/h)			PAS (mm)			Complications
	Pre	Post	Dx	Pre	Post	Dx	Pre	Post	Dx	Pre	Post	Dx	
Variation 1: Maxillary and mandibular advancement													
Rossi et al. 2019 [16]	33.5	11.9	−21.6 (64.48%)	NA	NA	NA	NA	NA	NA	9.21	16.5	+7.29	Nil
Brevi et al. 2011 [18]	24.2	7	−17.7 (73.14%)	8	8	0	32	5	−28	7	10	+3	NA
	87	14	−73 (83.90%)	13	0	−13	84	9	−75	8	15	+7	NA
Ronchi et al. 2010 [17]	70	1	−69 (98.57%)	16	0	−16	NA	NA	NA	8	14	+6	Worsened aesthetics
	60	15	−45 (75%)	13	1	−12	NA	NA	NA	4	10	+6	NA
	71	10	−61 (85.92%)	12	2	−10	NA	NA	NA	5	12	+7	NA
Alsaty et al. 2021 [19]	21.2	0.7	−20.5 (96.70%)	12	3	−9	NA	NA	NA	NA	NA	NA	Nil
Li et al. 2000 [20]	44 ^	1 ^	−43 (97.72%)	NA	NA	NA	NA	NA	NA	5	9	+4	Re-operation due to insufficient fixation
	90 ^	9 ^	−81 (90%)	NA	NA	NA	NA	NA	NA	3	8	+5	Nil
	77 ^	10 ^	−67 (87.01%)	NA	NA	NA	NA	NA	NA	9	13	+4	Removal of mandibular implants due to localized irritation
Variation 2: Maxillary advancement with mandible auto-rotation													
Hoshijima et al. 2015 [21]	18.8	7.6	11.2 (59.6%)	NA	NA	NA	NA	NA	NA	NA	NA	NA	NA
Ishida et al. 2019 [22]	15.3	2.8	12.5 (81.70%)	NA	NA	NA	NA	NA	NA	NA	NA	NA	NA
Variation 3: Maxillary advancement and mandibular setback													
Abdelwahab et al. 2023 [23]	37.17 ± 35.77	11.81 ± 15.74	25.36 (68.23%) *p* = 0.41	10.23 ± 4.38	4.22 ± 3.07	6.01 *p* = 0.006	11.43 ± 11.40	5.44 ± 7.96	5.99 *p* = 0.828	NA	NA	NA	Nil
Lu et al. 2024 [24]	22.8	10.1	12.7 (55.7%)	NA	NA	NA	NA	NA	NA	NA	NA	NA	Nil

Legend: AHI = Apnea–Hypopnea Index, RDI or ^ = Respiratory Disturb Index, ODI = Oxygen Desaturation Index, PAS = Posterior Airway Space, NA = Not available, Dx = Difference Of, Nil = None reported.

## Data Availability

Data is contained within the article or Supplementary Material.

## Supplementary Materials

The following supporting information can be downloaded at: https://www.mdpi.com/article/10.3390/jcm15030935/s1, File S1: Search Terms.
