# Supplementary material for: Is Maxillomandibular Advancement Possible in Skeletal Class III Patients? A Scoping Review
Source: J Clin Med. 2026 Jan 23;15(3):935. doi: 10.3390/jcm15030935 (PMC12898098; doi:10.3390/jcm15030935)
Supplement: Supplementary file 1 [file jcm-15-00935-s001.zip › jcm-4100739-supplementary.pdf]

Supplementary Material File S1

PubMed

((maxillomandibular advancement) OR (maxillo-mandibular advancement) OR (modified maxillomandibular advancement) OR (modified maxillo-mandibular advancement) OR (Maxillary advancement) OR (Bimaxillary advancement) OR (Telegnathic surgery) OR (Bimaxillary rotation) OR (Counter clockwise rotation) OR (Sub-apical osteotomy) OR (Prognathism) OR (Progenia) OR (Class III) OR (CLASS 3) OR (Mandibular hyperplasia)) AND ((obstructive sleep apnea) OR (obstructive sleep apnoea) OR (Sleep disordered breathing) OR (OSA) OR (OSAS) OR (apnea hypopnea index) OR (AHI) OR (apnea-hypopnea index) OR (respiratory distress index) OR (oxygen desaturation index) OR (lowest oxygen saturation) OR (LSAT) OR (Sleep Apnea Syndromes [Mesh]) OR (Sleep Apnea, Obstructive [Mesh]))

((Maxillary expansion) OR (Palatal expansion) OR (MARPE) OR (SARPE) OR (MISMARPE)) AND ((obstructive sleep apnea) OR (obstructive sleep apnoea) OR (Sleep disordered breathing) OR (OSA) OR (OSAS) OR (apnea hypopnea index) OR (AHI) OR (apnea-hypopnea index) OR (respiratory distress index) OR (oxygen desaturation index) OR (lowest oxygen saturation) OR (LSAT) OR (Sleep Apnea Syndromes [Mesh]) OR (Sleep Apnea, Obstructive [Mesh]))

Embase

('maxillomandibular advancement'/exp OR 'maxillomandibular advancement' OR (maxillomandibular AND advancement) OR 'maxillo-mandibular advancement' OR ('maxillo mandibular' AND advancement) OR 'modified maxillomandibular advancement' OR (modified AND maxillomandibular AND advancement) OR 'modified maxillo-mandibular advancement' OR (modified AND 'maxillo mandibular' AND advancement) OR 'maxillary advancement'/exp OR 'maxillary advancement' OR (('maxillary'/exp OR maxillary) AND advancement) OR 'bimaxillary advancement' OR (bimaxillary AND advancement) OR 'telegnathic surgery' OR (telegnathic AND ('surgery'/exp OR surgery)) OR 'bimaxillary rotation' OR (bimaxillary AND ('rotation'/exp OR rotation)) OR 'counter clockwise rotation' OR (('counter'/exp OR counter) AND clockwise AND ('rotation'/exp OR rotation)) OR 'sub-apical osteotomy' OR ('sub apical' AND ('osteotomy'/exp OR osteotomy)) OR 'prognathism'/exp OR prognathism OR 'progenia'/exp OR progenia OR 'class iii' OR (class AND iii) OR 'mandibular hyperplasia' OR (mandibular AND ('hyperplasia'/exp OR hyperplasia))) AND ('obstructive sleep apnea'/exp OR 'obstructive sleep apnea' OR (obstructive AND ('sleep'/exp OR sleep) AND ('apnea'/exp OR apnea)) OR 'obstructive sleep apnoea'/exp OR 'obstructive sleep apnoea' OR (obstructive AND ('sleep'/exp OR sleep) AND ('apnoea'/exp OR apnoea)) OR 'sleep disordered breathing'/exp OR 'sleep disordered breathing' OR (('sleep'/exp OR sleep) AND disordered AND ('breathing'/exp OR breathing)) OR osa OR osas OR 'apnea hypopnea index'/exp OR 'apnea hypopnea index' OR (('apnea'/exp OR apnea) AND ('hypopnea'/exp OR hypopnea) AND ('index'/exp OR index)) OR ahi OR 'apnea-hypopnea index'/exp OR 'apnea-hypopnea index' OR ('apnea hypopnea' AND ('index'/exp OR index)) OR 'respiratory distress index'/exp OR 'respiratory distress index' OR

(('respiratory'/exp OR respiratory) AND ('distress'/exp OR distress) AND ('index'/exp OR index)) OR 'oxygen desaturation index'/exp OR 'oxygen desaturation index' OR (('oxygen'/exp OR oxygen) AND ('desaturation'/exp OR desaturation) AND ('index'/exp OR index)) OR 'lowest oxygen saturation'/exp OR 'lowest oxygen saturation' OR (lowest AND ('oxygen'/exp OR oxygen) AND ('saturation'/exp OR saturation)) OR lsat)

Cochrane

((maxillomandibular advancement) OR (maxillo-mandibular advancement) OR (modified maxillomandibular advancement) OR (modified maxillo-mandibular advancement) OR (Maxillary advancement) OR (Bimaxillary advancement) OR (Telegnathic surgery) OR (Bimaxillary rotation) OR (Counter clockwise rotation) OR (Sub-apical osteotomy) OR (Prognathism) OR (Progenia) OR (Class III) OR (Class 3) OR (Mandibular hyperplasia)) AND ((obstructive sleep apnea) OR (obstructive sleep apnoea) OR (Sleep disordered breathing) OR (OSA) OR (OSAS) OR (apnea hypopnea index) OR (AHI) OR (apnea-hypopnea index) OR (respiratory distress index) OR (oxygen desaturation index) OR (lowest oxygen saturation) OR (LSAT))

LILACS

Maxillomandibular advancement
